# Bariatric surgery in obese patients with ventricular assist devices

**DOI:** 10.1186/s13104-020-05221-z

**Published:** 2020-08-14

**Authors:** Adrian daSilva-deAbreu, Bader Aldeen Alhafez, Yuhamy Curbelo-Pena, Carl J. Lavie, Hector O. Ventura, Juan Francisco Loro-Ferrer, Stacy A. Mandras

**Affiliations:** 1 John Ochsner Heart and Vascular Institute, Ochsner Clinic Foundation, New Orleans, LA USA; 2 The University of Queensland Ochsner Clinical School, Faculty of Medicine, The University of Queensland, New Orleans, LA USA; 3 Doctoral School, Universidad de Las Palmas de Gran Canaria, Las Palmas, Spain; 4 Department of Internal Medicine, The Ohio State University, Columbus, OH USA; 5 Service of General Surgery, Consorci Sanitari de L’Alt Penedes I Garraf, Barcelona, Spain

**Keywords:** Heart-assist devices, Ventricular assist devices, Bariatric surgery, Sleeve gastrectomy, Gastric bypass, Roux-en-Y gastric bypass, Obesity, Heart failure, Body mass index, Heart transplantation

## Abstract

**Objectives:**

Patients with end-stage heart failure (ESHF) treated with ventricular assist devices (VADs) tend to gain weight, which may prevent them from receiving heart transplantation (HT) if their body mass index (BMI) reaches ≥ 35 kg/m^2^. The objective was to synthesize all cases available in the literature and describe the most important outcomes of bariatric surgery (BS) in VAD patients, including BMI trends, reaching a BMI < 35 kg/m^2^, listing for HT, achieving HT, myocardial recovery, and mortality. These data were obtained for an individual participant data (IPD) meta-analysis and include available IPD for every case in the scientific literature describing VAD patients undergoing BS during VAD support with documented postoperative BMI (and time of measurement) during follow-up.

**Data description:**

These data include baseline, periprocedural, and long-term outcomes for the 29 patients meeting selection criteria. The composite outcome includes reaching a BMI < 35 kg/m^2^, listing for HT, receiving HT, and myocardial recovery, indicating significant BMI loss associated with major ESHF outcomes. As multiple centers are becoming more experienced in this field, the present data can be merged with their databases to form larger samples that will allow to perform further statistical analysis to identify outcome predictors and improve clinical protocols and outcomes.

## Objective

Patients with class II obesity (body mass index; BMI ≥ 35 kg/m^2^) have worse outcomes after heart transplantation (HT). Hence, the International Society for Heart and Lung Transplantation considers a BMI ≥ 35 kg/m^2^ to be a contraindication for HT [1], excluding many obese patients with end-stage heart failure (ESHF) from transplantation, which is the gold standard therapy for this condition.

An alternative therapy for obese patients with ESHF is ventricular assist device (VAD) implantation. The BMI cutoff for VAD implantation varies among different centers and is often higher than for HT. Based on this, one would expect obese patients to undergo VAD implantation, lose weight, and undergo HT once they achieve a BMI < 35 kg/m^2^. However, patients with BMIs < 35 kg/m^2^ tend to gain weight after VAD implantation [2], which may halt their candidacy for HT if they reach a BMI ≥ 35 kg/m^2^.

Few small cohort studies and case reports have shown promising results of bariatric surgery (BS) in obese patients with VADs [3–6]. Nevertheless, much of the postoperative weight (and BMI) trends remains unknown in this population. This is an important knowledge gap because VAD patients receive chronic anticoagulation and are already at very high risk of thromboembolic and bleeding events at baseline [7] which increases the risk of adverse events after surgeries. In consequence, only few large centers offer this intervention to a minority of obese patients with VADs.

Therefore, this systematic review and individual participant data (IPD) meta-analysis included available IPD for every case in the scientific literature describing the outcomes of VAD patients who underwent BS with documented postoperative BMI (and time of measurement) during follow-up [8]. The aim was to evaluate the most important outcomes after BS in VAD patients, including BMI trends, listing for HT, achieving HT, myocardial recovery, and mortality.

## Data description

A systematic search was conducted in PubMed, Embase, The Cochrane Library and ClinicalTrials.gov combining free keywords and official indexing terms for VADs and BS. Truncated terms were used when allowed by the engine. Additional searches were performed in Google Scholar and the online content of: The Journal of Heart and Lung Transplantation, Journal of Cardiac Failure, Obesity Surgery, Surgery for Obesity and Related Diseases, and every member of the journal groups: Journal of the American College of Cardiology, Circulation, and the European Heart Journal.

Retrieved references were transferred to EndNote X8 (Clarivate Analytics, Philadelphia, PA, USA) and Rayyan (Qatar Computing Research Institute, Doha, Qatar), and duplicates were removed. Studies of any design and publication type (abstracts, full article, registry with results…) were included if they contained IPD of postoperative BMIs of VAD patients undergoing BS. Two investigators worked in parallel and independently to select references, extract IPD, and assess the risk of bias in each study. Discrepancies were resolved by discussion.

Every study was classified according to the institution where they were conducted, publication year, sample size, number of patients that met selection criteria, publication type, and whether or not they met selection criteria, data available in Data file 1. Only non-duplicated IPD were obtained from reference(s) with largest sample size with available IPD for postoperative BMI. One patient’s IPD was included in a case report and a cohort study [9, 10], hence, such IPD were extracted from the most detailed reference [9]. When available, weight and additional anthropometric data were used to calculate BMI. All included studies had low risk of bias for the main outcome (postoperative BMI).

Data file 2 provides detailed data of the studies and baseline characteristics of patients that met selection criteria. Detailed BMI data over time, type of BS, length of follow-up, and most important clinical ESHF outcomes are available in Data file 3. These data evidence successful results of BS in obese VAD patients to achieve the composite outcome of a < 35 kg/m^2^, HT, listing for HT, or myocardial recovery.

Data file 4 displays graphical trends of the excess BMI lost over time after BS. Each line represents an individual patient and zero (0) corresponds to the time and baseline body mass index at the time of bariatric surgery which is the point of reference for BMI comparisons (excess BMI lost) during follow-up. This graph was developed with StataSE 14 (College Station, TX, USA). Data file 5 provides a list of the references cited in the tables.

As clinical practice and research interest in this field continues to grow, multiple centers of advanced heart failure and HT will potentially benefit from analyzing these data or merging these data with their own (single or multicenter) data to create a larger sample to perform more elaborate statistical analysis, such as regressions and subgroup analyses, to better understand the impact of BS in these obese patients with VADs and to identify predictors of major outcomes that ultimately translate into improvements in clinical practice and outcomes.

## Limitations

- As with every meta-analysis, there is a risk of publication bias of the available literature which could have biased our sample. To reduce this risk, we did not exclude abstracts and screened grey literature.
- There is also a risk of reporting bias, especially with case reports that did not report all major outcomes of interest.
- These data contain IPD of patients who already had VADs at the time of BS. Patients who underwent BS prior to VAD implantation or had both interventions done during the same encounter were excluded. Patients without available postoperative BMI data were excluded because the meta-analysis was centered on the BMI trends after BS. However, these data are very valuable as they provide better understanding and could even expedite future meta-analyses in the field by greatly reducing the amount of work required for data extraction and quality control. Furthermore, identifying the missing data from each reference allows to appreciate areas for improvement in the way we report studies and gaps of knowledge on this topic.

## Data Availability

The data described in this Data note can be freely and openly accessed on Mendeley Data under https://doi.org/10.17632/33m7sc3wnc.2 [11]. Please see Table 1 and references 8 and 11 for details and links to the data.

**Table 1:** Overview of data files/data sets

Label	Name of data file/data set	File types (file extension)	Data repository and identifier (DOI or accession number)
*Data file 1*	*Data file 1. References of patients with VAD undergoing bariatric surgery.xlsx*	*xlsx (or Excel)*	*Mendeley (*https://doi.org/10.17632/33m7sc3wnc.2*)*
*Data file 2*	*Data file 2. Baseline patient characteristics.xlsx*	*xlsx (or Excel)*	*Mendeley (*https://doi.org/10.17632/33m7sc3wnc.2*)*
*Data file 3*	*Data file 3. Perioperative and outcome data.xlsx*	*xlsx (or Excel)*	*Mendeley (*https://doi.org/10.17632/33m7sc3wnc.2*)*
*Data file 4*	*Data file 4. Trends of excess BMI after bariatric surgery in patients with VADs.png*	*png*	*Mendeley (*https://doi.org/10.17632/33m7sc3wnc.2*)*
*Data file 5*	*Data file 5. References for Data files 1–3.docx*	*docx (or Word)*	*Mendeley (*https://doi.org/10.17632/33m7sc3wnc.2*)* Overview of data files/data sets

## Abbreviations

BMI: Body mass index
BS: Bariatric surgery
ESHF: End-stage heart failure
HT: Heart transplantation
IPD: Individual participant data
VAD: Ventricular assist device
